# The Relationship Between Comprehensive OSCE Scores and GPA on the Readiness of Undergraduate Medical Students to Undergo Clinical Rotations

**DOI:** 10.21315/mjms-01-2025-009

**Published:** 2025-06-30

**Authors:** Ida Bagus Amertha Putra Manuaba, Made Violin Weda Yani, I Gusti Ayu Sri Darmayani, Putri Ayu Wulandari, Anak Agung Bagus Putra Indrakusuma, Ni Luh Putu Larasati Prabawaning Tyas, I Gede Putu Supadmanaba

**Affiliations:** 1 Department of Medical and Health Education, Faculty of Medicine, Udayana University, Denpasar, Bali, Indonesia; 2 Faculty of Medicine, Udayana University, Denpasar, Bali, Indonesia; 3 Department of Biochemical, Faculty of Medicine, Udayana University, Denpasar, Bali, Indonesia

**Keywords:** GPA, student readiness, comprehensive OSCE, clinical rotation

## Abstract

**Background:**

Objective Structured Clinical Examination (OSCE) is an essential clinical skills evaluation method used in the preclinical stage. OSCE increases students’ average readiness to practice the role of medical personnel. Good knowledge is expected to be directly proportional to clinical practice skills. The cumulative grade point average (GPA) is the parameter used to measure knowledge. Students are said to be ready to practice if they balance clinical skills, basic knowledge, and critical thinking which can be seen through comprehensive OSCE scores and student GPA. This study aims to examine the relationship between comprehensive OSCE scores and GPA on the readiness of undergraduate medical students to undergo clinical rotations at the Faculty of Medicine, Universitas Udayana.

**Methods:**

This study was conducted at the Faculty of Medicine, Universitas Udayana, from May to October 2024. Its subject was research on medical students who will undergo clinical rotations. The research instrument used was the Preparedness for Hospital Practice Questionnaire (PHPQ). Data analysis was carried out using SPSS for Windows version 25.0 software.

**Results:**

The mean age of respondents was 22.02 ± 0.835 years. Most respondents were female (61.1%) and international students (51.9%). The mean rating scale of the research respondents from the eight domains was 4–5, indicating that students’ readiness to undergo clinical rotations was adequate to adequate. Comprehensive OSCE scores and GPA have a significant relationship with the readiness of undergraduate medical students for clinical rotations, respectively, in the domains of collaboration, holistic care, and interpersonal skills. Conclusion: The results of this study can serve as a reference for evaluating the readiness of undergraduate medical students to undergo clinical rotations and for preparing appropriate learning strategies at the Faculty of Medicine, Universitas Udayana, particularly during the transition to clinical rotations.

## Introduction

A doctor is a professional with a licence to carry out health services. In the education series, general practitioners must undergo preclinical and clinical education. Preclinical education is education with most material in primary medical and scientific theory, some developments, and some basic clinical skills. Meanwhile, clinical education directly applies education by accumulating all theories and clinical skills that medical students possess (1).

One of the basic clinical skills evaluation methods used in the preclinical stage is the Objective Structured Clinical Examination (OSCE) (2). In the final preclinical semester, before entering the clinical stage, medical students will be tested with a comprehensive OSCE, which includes the accumulation of clinical skills taught from the early semester to the final semester of preclinical lectures following the competency standards listed in national guidelines (3). Several universities, especially the Faculty of Medicine, Universitas Udayana, use comprehensive OSCE as a parameter test for students to continue their studies to the next level, just like the OSCE in the previous semester.

Based on studies, knowledge related to basic medical science and good clinical skills is one of the major foundations that can influence readiness to undergo clinical rotations in co-assistants (4). Good knowledge is expected to be directly proportional to the practical skills that will be learned in the clinical rotation of the co-ass. The parameter commonly used to measure student knowledge is the grade point average (GPA) (5). This final value unit will describe the value of the student’s learning process during their studies, indicating their academic success or achievement (6).

Based on previous studies, it was found that OSCE experiences can improve students’ clinical skills (7). Studies found that good clinical skills are directly proportional to sound knowledge. This assessment can be seen through the student’s GPA (5). However, no study has assessed the correlation between GPA and comprehensive OSCE in viewing the picture of undergraduate students’ readiness to face co-ass clinical rotations. Thus, this study will examine the association between comprehensive OSCE scores and GPA on the readiness of undergraduate medical students to undergo co-ass clinical rotations at the Faculty of Medicine, Universitas Udayana.

## Methods

### Study Design

This study is an analytical study with a cross-sectional method that aims to determine the relationship between comprehensive OSCE scores and GPA and the readiness of undergraduate medical students to undergo clinical rotations. It will be conducted using an online survey given to students of the Faculty of Medicine, Universitas Udayana.

### Sample Selection

Sampling in this study was carried out by total sampling with a minimum sample size calculated using the Slovin formula and dropout criteria. Based on these calculations, the minimum sample size required for this study is 162 medical students.

### Data Collection

Data collection begins by collecting research subjects who meet the inclusion. The variables, namely comprehensive OSCE score and students’ GPA, were obtained from the academic data summary of the Faculty of Medicine, Universitas Udayana, which had been previously approved by the Deputy Dean for Education of the Faculty of Medicine, Universitas Udayana. Meanwhile, the readiness of undergraduate medical students to undergo clinical rotations was assessed using the Preparedness for Hospital Practice Questionnaire (PHPQ), which consists of 41 questions and eight domains (interpersonal skills, confidence, collaboration, management, science, prevention, holistic care, and self-directed learning). The data was collected in tabular form.

### Data Analysis

The data were analysed using SPSS for Windows, version 25.0 software. Univariate analysis of proportions was carried out to describe the basic characteristic variables of research subjects such as age, gender, class, comprehensive OSCE score, GPA, and student responses to PHPQ. Normality analysis of numerical data was carried out to determine the data distribution using the Kolmogorov-Smirnov test (total sample size > 100 people). The Levene homogeneity test was carried out to assess the variance of the data. The one-way ANOVA test was conducted to determine the difference in the mean comprehensive OSCE score and student GPA based on the level of student readiness to undergo clinical rotation interns if the data was normally distributed, and an alternative in the form of the Kruskal-Wallis test if the data was not normally distributed. The *P*-value is considered significant if *P* < 0.05.

## Results

A total of 162 students were involved in this study. Based on the data analysis that has been done, it was found that the average age of respondents was 22.02 ± 0.835 years. Most respondents were female (61.1%), and the number of males was 38.9%. Based on the class of respondents, 48.1% were in class A, and 51.9% were in class B. The median OSCE score of students was 88.8 (71–97.05), and students’ GPA was 3.765 (3.36–3.99). The average rating scale of the research respondents from the eight domains of PHPQ was in the range of 4–5, which means that the readiness of undergraduate medical students to undergo clinical rotations of co-ass was in the range of quite adequate to adequate. Most of the student’s readiness in the interpersonal skills, confidence, management, and science domains was fairly adequate. Meanwhile, students’ readiness in the domains of collaboration, prevention, holistic care, and self-directed learning is on an adequate scale. Complete data are presented in Table 1.

Based on the bivariate analysis, the comprehensive OSCE score had a significant relationship with undergraduate medical students’ readiness to undergo clinical rotations in the collaboration (*P* = 0.015) and holistic care (*P* = 0.016) domains (Table 2).

Based on the bivariate analysis, GPA values significantly relate to undergraduate medical students’ readiness to undergo clinical rotations in the interpersonal skills domain (*P* = 0.019) (Table 3).

## Discussion

### Characteristics of the Research Sample

Various aspects of each influence the readiness to undergo clinical rotations. Based on the characteristics obtained, the average age of the participants who became respondents in this study was 22 years. Previous studies have shown that the age of respondents does not influence perception or readiness when analysed individually (8). Based on gender, participants in this study were dominated by women. A study mentioned that men have better patient management skills and skills when dealing with patients, especially when performing practical procedures. Meanwhile, women tend to have a more humanistic side than men when interacting with patients. However, it was mentioned that there was no significant correlation between gender and the level of readiness to perform medical actions in clinical practice (8, 9).

PHPQ is the parameter needed in this study to assess preparedness with the results of all domains in the range of adequate to sufficient. Based on the research data, participants are professional in preparing themselves for clinical rotations. Readiness can be formed from all incorporated aspects and abilities and can increase self-efficacy. If students are ready for clinical rotations, they will become better doctors with better team integration. The association will continue to rotate circularly if each individual is aware of preparing themselves better (10).

Each PHPQ domain, namely interpersonal skills, confidence, collaboration, management, science, prevention, holistic care, and self-directed learning, is a component that can show readiness to undergo clinical rotations (10). The results of this study indicate that the readiness of students from the eight domains of the PHPQ questionnaire is in the range of 4–5, which means that the readiness of medical undergraduate students to undergo clinical rotations is adequate.

Interpersonal skills are one of the aspects that play an important role in the readiness of medical students when entering clinical rotations. A study published in *BMC Medical Education* (11) examined medical students at several universities. This study found that students with strong interpersonal skills demonstrated higher readiness in clinical rotations and exhibited lower anxiety levels compared to students with poor interpersonal skills. In addition, it was also found that there is a positive relationship between interpersonal skills and patient satisfaction and clinical performance, making this aspect essential in the medical curriculum (11, 12).

Readiness for the clinician rotation is also strongly influenced by confidence. Higher levels of confidence tend to improve readiness for dealing with unexpected clinical situations. Confidence built through clinical simulation and skills training has also been shown to improve readiness for dealing with real cases (13).

Furthermore, effective collaboration is one of the necessary team efficiencies and can reduce the risk of medical errors. Previous studies have suggested that good collaboration increases positive perceptions of clinical rotations and reduces stress due to mutual support between medical teams (14).

In addition to collaboration, management skills are required to manage time, priorities, and tasks during a busy schedule with complex cases during clinical rotations. Previous research mentioned that good management showed higher readiness to handle the clinical workload and balance personal and professional needs during rotations (15).

Furthermore, science or scientific knowledge supports readiness when entering clinical rotations, such as an in-depth understanding of basic medical sciences. Clinical students with good scientific knowledge perform better, especially when diagnosing and planning treatment. Studies show that with a good basic understanding, students can participate more effectively in clinical discussions and respond to emergencies (16, 17).

After that, prevention is an understanding of risk factors, health education, and interventions that can prevent or suppress disease progression. Understanding the concept of prevention during clinical rotations will support student readiness in educating patients. In addition, a holistic approach plays an important role in the readiness of students who will undergo clinical rotations because holistic care refers to efforts to see patients as whole individuals and not only focus on symptoms or diagnoses. Previous research suggests that medical students trained in a holistic approach show higher readiness in dealing with patients during clinical rotations and can identify complex patient needs with more comprehensive treatment planning (18).

The last aspect is self-directed learning. In a study conducted by Roberts et al. (19), SDL’s competence in medical students who will undergo clinical states has increased. This condition is in line with the research results of this study. This component is one of the important things in clinical rotation readiness because SDL is a competency in considering evidence-based care to patients so that it can improve the quality of learning in clinical rotations (19).

### The Relationship of Comprehensive OSCE Score and GPA to the Readiness of Undergraduate Medical Students to Undergo Co-ass Clinical Rotation

Studies that directly assess the association between GPA and comprehensive OSCE on the readiness of undergraduate medical students to undergo clinical rotation co-ass have yet to be conducted. Therefore, the results of this study can be used as one of the references for academic factors that can affect readiness in undergoing clinical rotation co-ass. The results of this study indicate that comprehensive OSCE scores and GPA are significantly associated with undergraduate medical students’ readiness for clinical rotation co-ass, respectively, in the domains of collaboration, holistic care, and interpersonal skills.

The results of this study align with those of Purnamasari and Setyawan (4), which stated that the knowledge factor (*P* = 0.04) influences students’ readiness to undergo clinical education. Students are considered ready to practice if they have basic knowledge and special practice skills to provide optimal care and management suggestions for patients (4). Gallagher et al.’s (20) study showed an association between student grades and student performance during clinical rotations. The study stated that the association between student grades and performance during clinical rotations provides valid evidence to recommend written assignments using notes to measure students’ clinical reasoning (20).

The OSCE was found to significantly increase the average readiness of students to practice their role as healthcare providers (*P* = 0.03). It is because the OSCE experience improves the clinical skills perceived by students (7). Therefore, students ready to undergo clinical rotation co-ass generally have good OSCE scores because they are skilled in providing care to mannequins and standard patients, whose skills will later be applied to real-life patients. Ginting and Yulfi’s (21) research shows that knowledge and skills during the preclinical period are essential factors contributing to the excellent perception of students’ readiness to undergo clinical rotation co-ass. Students’ ability to implement the knowledge gained during preclinical education affects their’ perceptions of readiness for clinical clerkships (21).

Comprehensive OSCE is essential in students’ readiness to undergo clinical rotation co-ass. Students are evaluated to provide complete management and interact with patients, even using standard patients. It follows one of the principles of patient-based learning, namely early clinical exposure (ECE). ECE helps students improve their academic, clinical, and communication skills to become more confident. ECE planning can be done in various situations using log books, textbooks, case reports, and computer devices (22).

OSCE is instrumental in preparing students before being placed in clinical practice, which requires high professionalism toward patients (23). Therefore, OSCE scores can reflect students’ ability to manage patients, indicating their readiness to follow clinical practice. Students with higher academic performance tend to get higher scores on clinical rotations (24). This indicates that GPA plays a role in the readiness of the clinical rotation of interns as an indicator of student knowledge regarding understanding patient health problems.

This study did not evaluate factors that could affect the association between GPA and comprehensive OSCE on readiness for co-ass clinical rotation. Other factors, such as student learning environment, learning motivation, conduciveness, and mental health, were not evaluated as confounding factors in this study. This study was an initial study that examines topics related to academic achievement on readiness for co-ass clinical rotation. It needs to be developed for use as a material to evaluate student learning methods.

## Conclusion

The mean rating scale of the respondents’ PHPQ domain is in the range of 4–5, which means that the readiness of medical undergraduate students to undergo clinical rotations is in the range of adequate to adequate. It was also found that comprehensive OSCE scores and GPA had a significant relationship with the readiness of undergraduate medical students for clinical rotations, respectively, in the domains of collaboration, holistic care, and interpersonal skills.

## Figures and Tables

**Table 1:** Characteristics of research subjects

Variables	Sample (*N* = 162)	

	*n*	%
**Age (Mean ± SD)**	22.020 ± 0.835	
**Gender**		
Male	63	38.9
Female	99	61.1
**Class**		
A (regular)	78	48.1
B (international)	84	51.9
**OSCE Score (Median [Min–Max])**	88.8 (71.00–97.05)	
**GPA (Median [Min–Max])**	3.765 (3.36–3.99)	
**PHPQ Domain**	**Average rating scale**	**95% CI**
Interpersonal skills	4.25	3.99–4.52
Confidence	4.44	4.22–4.66
Collaboration	4.53	4.30–4.75
Management	4.51	4.31–4.72
Science	4.49	4.27–4.71
Prevention	4.77	4.58–4.95
Holistic care	4.77	4.58–4.95
Self-directed learning	4.69	4.48–4.91
**PHPQ domain (scale 1–6)**		
**Interpersonal skills**		
Very inadequate	3	1.9
Inadequate	6	3.7
Somewhat inadequate	12	7.4
Moderately adequate	66	40.7
Adequate	57	35.2
Very adequate	18	11.1
**Confidence**		
Very inadequate	0	0.0
Inadequate	0	0.0
Somewhat inadequate	12	7.4
Moderately adequate	66	40.7
Adequate	63	38.9
Very adequate	21	13.0
**Collaboration**		
Very inadequate	0	0.0
Inadequate	0	0.0
Somewhat inadequate	12	7.4
Moderately adequate	63	40.7
Adequate	66	38.9
Very adequate	21	13.0
**Management**		
Very inadequate	0	0.0
Inadequate	3	1.9
Somewhat inadequate	6	3.7
Moderately adequate	81	50.0
Adequate	54	33.3
Very adequate	18	11.1
**Science**		
Very inadequate	0	0
Inadequate	0	0
Somewhat inadequate	9	5.6
Moderately adequate	75	46.3
Adequate	51	31.5
Very adequate	27	16.7
**Prevention**		
Very inadequate	0	0.0
Inadequate	0	0.0
Somewhat inadequate	3	1.9
Moderately adequate	45	27.8
Adequate	81	50.0
Very adequate	33	20.4
**Holistic care**		
Very inadequate	0	0.0
Inadequate	0	0.0
Somewhat inadequate	3	1.9
Moderately adequate	54	33.3
Adequate	72	44.4
Very adequate	33	20.4
**Self-directed learning**		
Very inadequate	0	0.0
Inadequate	3	1.9
Somewhat inadequate	3	1.9
Moderately adequate	54	33.3
Adequate	66	40.7
Very adequate	36	22.2

Rating scale: 1 (very inadequate preparedness) – 6 (very adequate preparedness)

**Table 2:** The relationship between comprehensive OSCE scores and undergraduate medical students’ readiness to undergo internship clinical rotations

Domain		Median (Min–Max)	*P*
Interpersonal skills	Very inadequate	86.10 (86.10–86.10)	0.509
	Inadequate	89.20 (89.00–89.40)	
	Somewhat inadequate	84.00 (71.00–90.00)	
	Moderately adequate	88.73 (78.00–97.00)	
	Adequate	89.00 (71.75–97.05)	
	Very adequate	87.50 (73.25–93.00)	

Confidence	Very inadequate	0.00	0.790
	Inadequate	0.00	
	Somewhat inadequate	87.89 (78.00–96.65)	
	Moderately adequate	88.50 (71.00–97.00)	
	Adequate	89.00 (71.75–97.05)	
	Very adequate	88.00 (82.00–93.00)	

Collaboration	Very inadequate	0.00	0.015*
	Inadequate	0.00	
	Somewhat inadequate	82.04 (71.00–89.98)	
	Moderately adequate	89.00 (78.00–93.88)	
	Adequate	90.16 (71.75–97.05)	
	Very adequate	88.00 (82.00–93.00)	

Management	Very inadequate	0.00	0.355
	Inadequate	86.08 (86.08–86.08)	
	Somewhat inadequate	83.99 (78.00–89.98)	
	Moderately adequate	88.60 (71.00–96.65)	
	Adequate	90.25 (71.75–97.05)	
	Very adequate	87.50 (82.00–90.775)	

Science	Very inadequate	0.00	0.140
	Inadequate	0.00	
	Somewhat inadequate	86.075 (78.00–90.00)	
	Moderately adequate	89.00 (71.00–96.65)	
	Adequate	85.00 (71.75–97.00)	
	Very adequate	89.55 (82.00–97.05)	

Prevention	Very inadequate	0.00	0.050
	Inadequate	0.00	
	Somewhat inadequate	78.00 (78.00–78.00)	
	Moderately adequate	88.00 (78.00–96.65)	
	Adequate	89.70 (71.00–97.05)	
	Very adequate	88.00 (73.24–93.00)	

Holistic care	Very inadequate	0.00	0.016*
	Inadequate	0.00	
	Somewhat inadequate	78.00 (78.00–78.00)	
	Moderately adequate	88.00 (71.00–96.65)	
	Adequate	88.80 (71.75–97.05)	
	Very adequate	89.55 (82.00–93.00)	

Self-directed learning	Very inadequate	0	0.063
	Inadequate	86.08 (86.08–86.08)	
	Somewhat inadequate	78.00 (78.00–78.00)	
	Moderately adequate	88.20 (78.00–93.875)	
	Adequate	89.70 (71.00–97.00)	
	Very adequate	88.80 (82.00–97.05)	

* The *P*-value is significant (*P* < 0.05)

**Table 3:** The relationship between GPA and readiness of undergraduate medical students to undergo clinical internship rotation

Domain		Median (Min–Max)	*P*
Interpersonal skills	Very inadequate	3.85 (3.85–3.85)	0.019*
	Inadequate	3.90 (3.87–3.93)	
	Somewhat inadequate	3.655 (3.60–3.81)	
	Moderately adequate	3.79 (3.54–3.95)	
	Adequate	3.71 (3.36–3.99)	
	Very adequate	3.85 (3.62–3.92)	

Confidence	Very inadequate	0.00	0.647
	Inadequate	0.00	
	Somewhat inadequate	3.84 (3.60–3.90)	
	Moderately adequate	3.79 (3.54–3.93)	
	Adequate	3.74 (3.36–3.99)	
	Very adequate	3.81 (3.62–3.95)	

Collaboration	Very inadequate	0.00	0.110
	Inadequate	0.00	
	Somewhat inadequate	3.725 (3.60–3.85)	
	Moderately adequate	3.81 (3.54–3.95)	
	Adequate	3.82 (3.36–3.99)	
	Very adequate	3.70 (3.62–3.91)	

Management	Very inadequate	0.00	0.267
	Inadequate	3.85 (3.85–3.85)	
	Somewhat inadequate	3.715 (3.60–3.83)	
	Moderately adequate	3.75 (3.54–3.93)	
	Adequate	3.835 (3.36–3.99)	
	Very adequate	3.69 (3.62–3.92)	

Science	Very inadequate	0.00	0.101
	Inadequate	0.00	
	Somewhat inadequate	3.69 (3.60–3.85)	
	Moderately adequate	3.81 (3.56–3.93)	
	Adequate	3.75 (3.36–3.99)	
	Very adequate	3.86 (3.62–3.95)	

Prevention	Very inadequate	0.00	0.066
	Inadequate	0.00	
	Somewhat inadequate	3.60 (3.60–3.60)	
	Moderately adequate	3.82 (3.54–3.93)	
	Adequate	3.75 (3.36–3.99)	
	Very adequate	3.81 (3.62–3.96)	

Holistic Care	Very inadequate	0.00	0.078
	Inadequate	0.00	
	Somewhat inadequate	3.60 (3.60–3.60)	
	Moderately adequate	3.82 (3.54–3.93)	
	Adequate	3,725 (3.36–3.99)	
	Very adequate	3.81 (3.62–3.95)	

Self-directed learning	Very inadequate	0.00	0.098
	Inadequate	3.85 (3.85–3.85)	
	Somewhat inadequate	3.60 (3.60–3.60)	
	Moderately adequate	3.76 (3.54–3.92)	
	Adequate	3,785 (3.36–3.99)	
	Very adequate	3,785 (3.62–3.96)	

* The *P*-value is significant (*P* < 0.05)
